# The Microbiome of Nurdles: Life on the Primary Microplastics of the Texas Gulf Coast

**DOI:** 10.17912/micropub.biology.002273

**Published:** 2026-07-29

**Authors:** Vibha Annaswamy, Michelle Mikesh, Kasia Dinkeloo

**Affiliations:** 1 Freshman Research Initiative, The University of Texas at Austin, Austin, TX, United States; 2 The University of Texas at Austin, Austin, TX, United States

## Abstract

Nurdles are small, pre-production plastic pellets. Globally, nurdles are the second largest source of microplastic pollution due to release during manufacture and transport. As these nurdles persist in the environment, they undergo weathering—a process that significantly increases surface area and colonization by microbes. To gain an understanding of the composition of the microbiome found on nurdles, full-length 16S targeted metagenomic sequencing was performed on DNA extracted from nurdles collected from the Texas Gulf Coast. Sequencing data showed a greater amount and diversity of microbes found to be associated with nurdles than with the sand from which the nurdles were collected.

**Figure 1. Nurdles collected from the Texas Gulf Coast Harbor a Unique Microbiome:** Nurdles collected from the wrack line (A). Rinsed nurdles imaged via stereomicroscopy (B). Scanning Electron Microscopy of the surface of a nurdle showing microbial cells (C). Relative abundance of top nine genera represented in the targeted metagenomic sequencing data set of nurdles compared to sand.

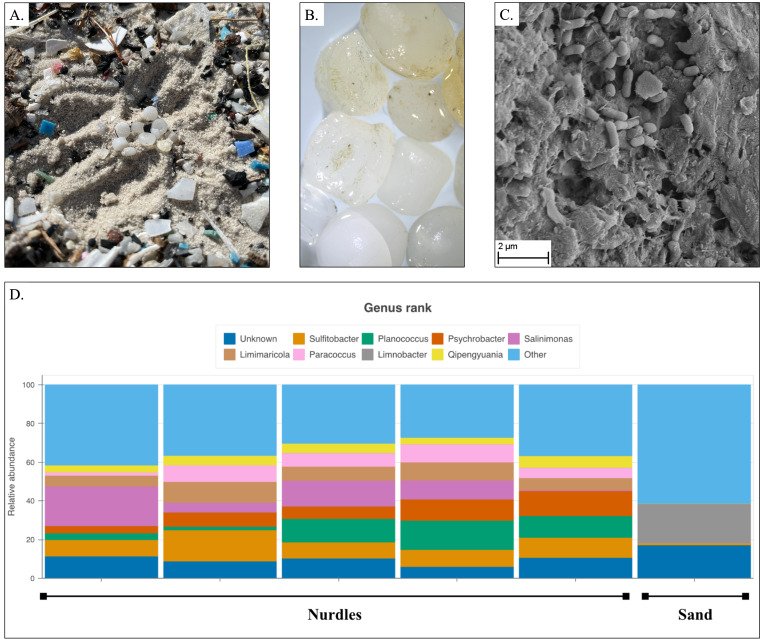



## Description

Microplastics present an immense crisis for the marine environment. Nurdles represent the second largest source of microplastic emissions globally, with over 445,000 tons of nurdles discharged into the environment yearly (Sewwandi et al. 2023). These small, pre-production plastic pellets are typically composed of a single polymer, but may contain chemical additives (Mato et al. 2001). They are spilled or released into the environment during transport from production factories to manufacturing facilities, often discharging directly into the ocean from shipping accidents or into secondary waterways from transport via train or truck (Sewwandi et al. 2023). Large numbers of nurdles have been documented on the Texas Gulf Coast since 2018, with some spill events resulting in an estimated 300,000 to 1 million nurdles per mile present for at least 24 miles of Mustang and North Padre Islands (Tunnell et al. 2020) (Fig 1A). As nurdles spend time in the environment, they are exposed to heat, moisture, sunlight, and physical forces. All of these elements contribute to a process known as weathering, which can result in changes to plastics such as increases in surface area from cracking or pitting, color changes, creation of chemical by-products from polymer degradation, and a decrease in mechanical strength (Andrady 2022) (Fig 1B).

The effects of nurdles in the environment have been investigated with regard to ingestion by sea life and incidence in different habitats, but little is known about how microbes may interact with or act upon these primary microplastics. Since these pellets are resilient and buoyant, they serve as ideal surfaces for the formation of biofilm and accumulation of various microbes as they travel through the environment over long periods of time (Wright et al. 2020, Oberbeckmann et al. 2015). The nurdles we collected showed visual evidence of diverse microbial colonization (Fig 1C). Several studies have shown that pieces of plastic debris from marine environments collect unique microbiomes (Zettler et al. 2013) and that nurdles may host human pathogens (Rodrigues et al. 2019), but there is no published data available on the full composition of the microbiome associated with Gulf Coast nurdles.

While nurdles account for an increasing percentage of primary microplastic pollution in many environments, they lack any sort of hazard classification that might aid in their mitigation or clean-up support from U.S. government sources. Studies have been conducted on their polymer composition, showing that a majority of nurdles recovered from Gulf Coast beaches are made of polyethylene (PE) or polypropylene (PP) (Jiang et al. 2022). Most of these nurdles likely originate from the Texas Gulf Coast (Cisco et al. 2025). Further studies demonstrate an association of toxic chemicals with nurdles as they weather and travel throughout the environment (Jiang et al. 2021). Understanding the microbiome composition of nurdles will provide a foundation for further research on the impact of this type of pollution. Future studies can examine potential hazards against human health, aquaculture, and agriculture, and prospect for the discovery and characterization of novel marine bacteria with an eye towards plastic degradation, spill-origin tracking, or other useful characteristics.

To gain this initial understanding of the nurdle microbiome, targeted metagenomic sequencing was conducted on microbial DNA isolated from groups of nurdles collected from the Texas Gulf Coast. Genomic DNA extracted from the groups of nurdles ranged from 32.6-133 ng. PCR amplification and purification of the full-length 16S region yielded 49-204 ng of amplicon DNA. Genomic DNA was also extracted and amplified from sand collected at the sampling location, yielding 272 ng of genomic DNA and 97.4 ng of purified 16S amplicon. This suggests a roughly equivalent microbial presence in both sand and nurdle samples. Samples were normalized such that the pooled library included 10 ng of amplicon DNA from each sample, and full-length 16S metagenomic sequencing was completed using the Oxford Nanopore MinION platform. The relative abundances of microbial genera identified from nurdle-associated microbial DNA were compared with sand-associated microbial DNA (Fig 1D).

Sequencing results were analyzed using the Minimap2 pipeline via EPI2ME to generate a rarefied abundance table at the genus rank. We saw a greater variety of microbial genera associated with nurdles that were notably distinct from the sand sample, suggesting that the nurdle microbiome is unique to its plastic environment. Of the top nine genera represented in nurdle samples, only one was found to be shared with the sand sample ( *Sulfitobacter* ), which was represented as 8-16% of reads for nurdle samples compared to 0.8% of reads from the sand sample (Fig 1D). The average effective number of species for nurdle samples was 65, compared with 33 for the sand sample. This trend in diversity was reflected in the Shannon’s diversity index as well, with the nurdle samples scoring an average of 4.17, compared to 3.5 for sand.

This data shows a great diversity of microbes associated with the surface of nurdles, some of which may also contribute to a “hazard” classification for the marine pollutant: several genera of pathogenic bacteria were found, with *Vibrio* species displayed in the highest abundance and found in all samples. Members of the genus Vibrio can be pathogens of fish and humans (Vandeputte et al. 2024). Other pathogenic genera represented in the nurdle data set are *Shigella* , *Escherichia, Tenacibaculum, Flavobacterium,* and *Salmonella* (The et al. 2016, Fernández-Álvarez and Santos 2018, Lee et al. 2023). Further investigation into the presence of these pathogenic genera on nurdles could impact guidelines for recreation or aquaculture in areas with high nurdle pollution.

Overall, we conclude that nurdles, like other marine microplastics, carry a unique microbiome that is distinct from the sand upon which they were collected. This microbiome is composed of a diverse set of both known and unknown microbes. Further studies should be conducted to better understand the full scale of the metagenome surrounding the nurdle and the roles that these bacteria play for their “host” and adjacent environments. The presence of these microbes gives rise to additional questions regarding seasonality, influence of polymer composition or weathering status, and location of nurdles.

## Methods

Nurdle Collection

Nurdles and sand were collected from the wrack line of the Horace Caldwell Pier beach in Port Aransas, TX, USA. They were harvested using forceps and rinsed lightly with 1X PBS buffer before storage. Nurdles were placed in 50 mL conical tubes and transported on ice to the lab. They were stored at 4°C until DNA extraction.

DNA Extraction

Aliquots of 50 nurdles per sample (or 100 mg of sand) were rinsed in 1X PBS and placed in 15 mL conical tubes. Samples were vortexed for 15 minutes with 1 mL of CD1 lysis buffer from the Qiagen DNeasy Powersoil Pro kit (CAT#47014), and DNA extraction proceeded according to the manufacturer’s protocol. Samples were eluted in 50 µL of elution buffer and stored at -20°C. DNA concentrations was measured using a QuBit Fluorometer with the HS dsDNA kit (CAT# Q32851).

Microscopy

Stereomicroscopy was performed with a ZEISS Stemi 508 stereo microscope with an Axiocam 208 color camera. Scanning electron microscopy (SEM) was performed to analyze microbes on the surface of nurdles. Briefly, sample preparation entailed: fix with 0.1 M sodium cacodylate, 2% glutaraldehyde, and 2% paraformaldehyde, wash with 0.1 M sodium cacodylate, and stain using 0.1 M sodium cacodylate, 1% osmium tetroxide, and 1% tannic acid. Samples dehydrated with increasing concentrations of ethanol and exchanged to hexamethyldisilazane before air drying. Samples were then mounted to stubs, sputter coated with platinum/palladium, and imaged at accelerating voltage using a Zeiss Scanning Electron Microscope.

16S Targeted Metagenomic Sequencing

Microbial DNA extracted from nurdles and sand was used to prepare samples for metagenomic sequencing using the 16S Barcoding Kit 24 V14 (ONT, SQK-16S114-24). PCR amplification and barcoding was completed with 10 ng template DNA, using Hot-Start Q5 2X Master Mix (New England Biolabs, CAT# M0494S). Initial denaturation at 98°C was followed by 25 cycles of 20 s at 98°C, 30 s at 55°C, 30 s at 72°C, and a final extension step of 5 min at 72°C. Purification of amplicons was performed using the AMPure XP Beads (Beckman Coulter) as per ONT’s instructions. Samples were measured via Qubit Fluorometer (Life Technologies) and pooled in an equimolar ratio to a total of 150 ng. Prepared libraries were run on a MinION Mk1B sequencer using a FLO-FLG114 flow cell. MINKNOW v24.06.8 with high-accuracy model v4.3.0, 400 bps basecalling was used for data acquisition.

Metagenomic Sequencing Data Analysis

Data analysis was performed on the EPI2ME platform using the standard wf-metagenomics nextflow workflow (v2.11.0) and Minimap2 (v2.26-r1175) with the following parameters: 90% minimum percent identity, 90% minimum reference coverage, ncbi_16s_18s reference database.
